# Structural Damage Detection Using PZT Transmission Line Circuit Model

**DOI:** 10.3390/s24227113

**Published:** 2024-11-05

**Authors:** Jozue Vieira Filho, Nicolás E. Cortez, Mario De Oliveira, Luis Paulo M. Lima, Gyuhae Park

**Affiliations:** 1 School of Engineering, Sao Paulo State University (Unesp), São João da Boa Vista 13876-750, SP, Brazil; jozue.vieira@unesp.br; 2 Department of Electrical Engineering, Federal University of Mato Grosso, Cuiabá 78060-900, MT, Brazil; ncortez@ufmt.br; 3 College of Engineering, Birmingham City University, Birmingham B4 7XG, UK; 4 Active Structures & Dynamics Laboratory, School of Mechanical Engineering, Chonnam National University, Gwangju 61186, Republic of Korea; luispauloml@gmail.com (L.P.M.L.); gpark@chonnam.ac.kr (G.P.)

**Keywords:** crosstalk effect, SHM, electromechanical impedance, structural monitoring, piezoelectric transducers

## Abstract

Arrangements of piezoelectric transducers, such as PZT (lead zirconate titanate), have been widely used in numerous structural health monitoring (SHM) applications. Usually, when two or more PZT transducers are placed close together, significant interference, namely crosstalk, appears. Such an effect is usually neglected in most SHM applications. However, it can potentially be used as a sensitive parameter to identify structural faults. Accordingly, this work proposes using the crosstalk effect in an arrangement of PZT transducers modeled as a multiconductor transmission line to detect structural damage. This effect is exploited by computing an impedance matrix representing a host structure with PZTs attached to it. The proposed method was assessed in an aluminum beam structure with two PZTs attached to it using finite element modeling in OnScale^®^ software to simulate both healthy and damaged conditions. Similarly, experimental tests were also carried out. The results, when compared to those obtained using a traditional electromechanical impedance (EMI) method, prove that the new approach significantly improved the sensitivity of EMI-based technique in SHM applications.

## 1. Introduction

Transducers based on piezoelectric effects have been extensively used in structural health monitoring (SHM) in different fields of engineering such as civil, mechanical, naval, aerospace, and so on. Most of the SHM techniques that use piezoelectric transducers can be divided into two broad areas: those based on electromechanical impedance (EMI) [1,2,3] and those based on measuring mechanical vibrations and wave propagation [4,5,6,7]. In both cases, patches of piezoelectric materials, most commonly lead zirconate titanate (PZT), are attached to the structure of interest and used as sensors, actuators, or both. In EMI techniques, the same patch is used simultaneously as both a sensor and actuator, while in mechanical vibration and wave propagation approaches, the same can be carried out but not simultaneously [8].

The basic principle of EMI-based techniques is to monitor the structure by measuring the electrical impedance of each PZT transducer bonded to the structure. Because of the coupling of PZT with the structure, the measured impedance can be used to assess the structure’s health state and detect damage. The signals obtained from the PZTs can then be analyzed in time or frequency by a variety of methodologies [9,10,11]. Even though EMI-based techniques in SHM are well established, they are still evolving. For example, there have been many studies on the aspects that degrade PZT performance, such as environmental, temperature, and bonding effects [12,13]. Another concern is that this approach has been the most successful when monitoring small areas around PZT. Although there are examples of using the transfer impedance between two PZTs in SHM [14], this subject still has plenty of room to be explored.

The electromagnetic coupling between conductors of a multiconductor transmission line is usually referred to as crosstalk, and there is vast amount of the literature on it. For example, in [15], the author presented a methodology to compute crosstalk in a multiconductor transmission line focused on low-frequency power systems. Adding to their results, in [16], a complete modal analysis to compute crosstalk in multiconductor coupled systems was presented, allowing a transmission system with N-coupled conductors to be transformed into N single isolated lines with a reference conductor. A similar work was presented in [17], where the authors used modal analysis and considered the length of the transmission line. The interference between cables and parallel wires has also been studied in other contexts, such as aeronautics and electronics. For example, in [18], the authors proposed a methodology to assess the effect of crosstalk in the presence of lossy ground planes in aircraft applications, and in [19], a technique to reduce the coupling effects among closely spaced microstrip lines was presented, which is an issue in high-density printed circuit boards. Crosstalk has also been studied in arrays of closely spaced piezoelectric elements [20,21,22]. In most applications, crosstalk is undesirable and must be mitigated [23,24,25,26,27,28,29]. However, there is a lack of studies that employ the concept of crosstalk effects in piezoelectric elements to enhance the sensitivity of EMI-based methods in SHM applications.

Although modeling PZTs as equivalent electric circuits is not a novelty, this has been mostly applied to design, simulate, and optimize PZT transducers according to a specific application, and it is mostly neglected in the SHM field. A new approach for modeling the interaction of two PZTs attached to a mechanical structure was proposed in [30], with a multiconductor transmission line model for PZT transducers based on controlled sources of current and voltage. The proposed technique was tested by simulating the electric circuit of an arrangement of two PZTs applied for structural damage detection, and although the results showed the viability of the technique, no experimental tests were carried out, and the simulation was limited to certain conditions.

Unlike previous works, this paper proposes a new approach in which PZTs are modeled as power transmission lines. This allows us to accurately define the interaction between PZTs located at a realistic distance from each other. Simulations and experimental tests were carried out to validate the proposed method. The results obtained for modeling PZTs as transmission lines demonstrated more sensitivity to damage identification compared to traditional EMI-based techniques, creating a new foundation on which to improve techniques for SHM applications.

The remainder of this paper is organized as follows. Firstly, the basic theory for modeling PZT as a transmission line and defining the mutual impedance is introduced. Secondly, the proposed method based on mutual impedance and self-impedance is presented. Next, the results from the simulations and experimental tests are presented and compared with the results based on the traditional EMI technique. Finally, this paper concludes with highlighting remarks on the proposed approach.

## 2. Theory and Background

The piezoelectric effect can be generally defined as the conversion of mechanical energy into electrical energy, the direct effect, or the conversion of electrical energy into mechanical energy, the inverse effect. The constitutive equation of piezoelectric materials, which relates its physical properties, such as the elastic and dielectric constants, to its electrical potential and internal stress, has been analyzed in different works [2,3,31]. The first models were proposed by Mason in 1942 using a lumped equivalent circuit [31], and in 1961, Redwood modified Mason’s model to obtain the transient response of a transducer [32]. In 1970, Krimholtz et al. [32] proposed a new equivalent circuit based on a frequency-dependent network. These cited works used an electric transformer as a common element, which led to negative impedances. This drawback was overcome by the model proposed by Leach in 1994 [33], by replacing the transformers with controlled current and voltage sources. The proposed model in this paper is based on Leach’s work.

### 2.1. The Piezoelectric Element as a Transmission Power Line

The following analysis is based on the pioneer work proposed by Leach [33]. It assumes all electric and mechanical variables (current, voltage, displacement, and speed) are Laplace transforms of the original time functions, simplifying the analysis without sacrificing generality.

**Figure 1:**
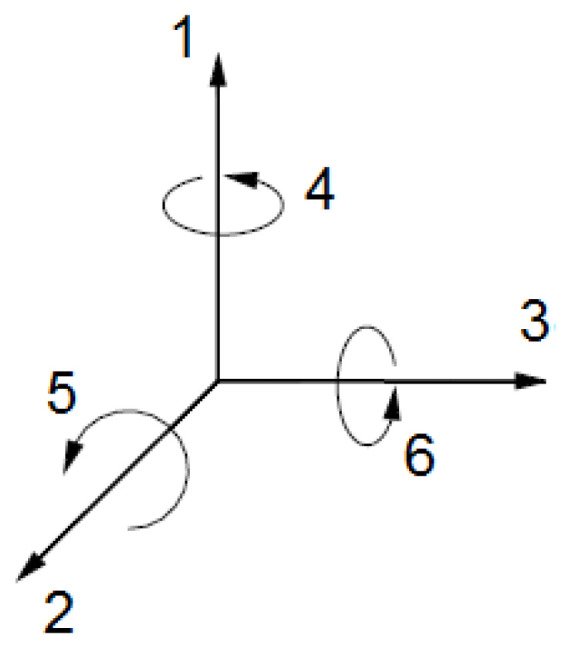
Stresses directions in piezoelectric transducers.

From a physical point of view, a piezoelectric ceramic can experience six different types of mechanical stresses: three longitudinal in the coordinate axes directions and three transverses or around the coordinate axes, as shown in Figure 1.

**Figure 2:**
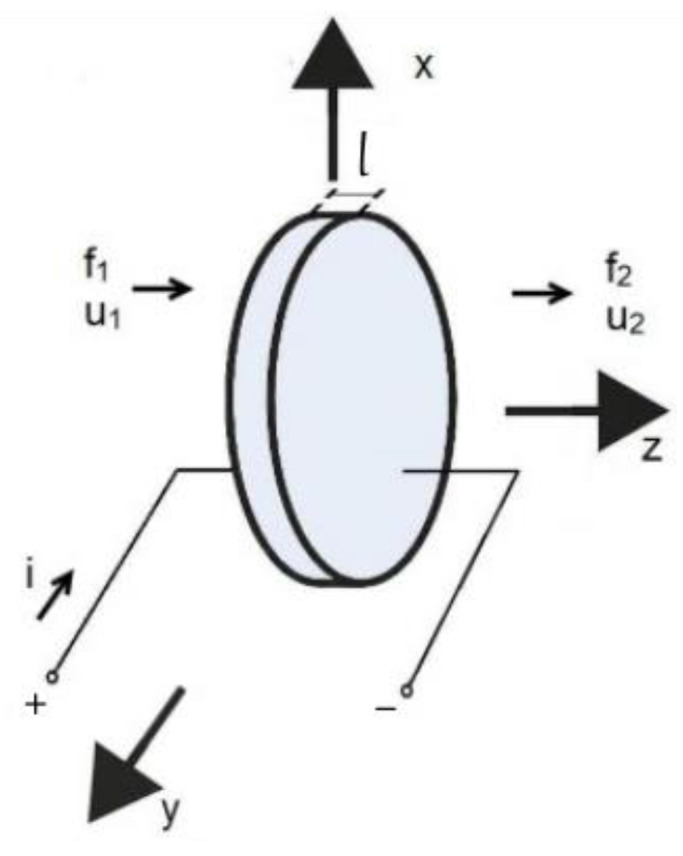
PZT’s thickness mode diagram.

Considering a small PZT patch bonded to a structure, which is a typical application for damage detection in SHM systems, z-axis polarization direction must be considered since it represents the thickness mode of the piezoelectric. Figure 2 depicts the diagram of the thickness mode of a piezoelectric disc transducer in which l is the dimension in z-axis. $f_{1}$ and $f_{2}\;$ are the external forces applied to the face of the piezoelectric in the z-axis direction, $u_{1}$ and $u_{2}\;$ are mechanical wave velocities, and i is the electric current flowing through it.

According to [31,32,34], ρ is the transducer density, $A_{z}$ is its perpendicular area to the z-axis, c is the relative elastic constant, h is the piezoelectric constant, and ε is the material’s permittivity constant. The equations that govern the wave propagation in the z-axis direction (thickness mode), are given as follows:

(1) $$\frac{df}{dz}=-\rho A_{z}su,$$

(2) $$c\frac{d\zeta }{dz}=-\frac{1}{A_{z}}f+hD,$$

(3) $$E=-h\frac{d\zeta }{dz}+\frac{1}{\varepsilon }D,$$

where s is Laplace domain’s complex frequency, E is the electric field intensity, D is the electric flow density, which is given by $D=q/A_{z}=i/\left(sA_{z}\right)$ with q=i/s being the electric charge in the transducer, and ζ is the wave-particle displacement given by ζ=u/s.

**Figure 3:**
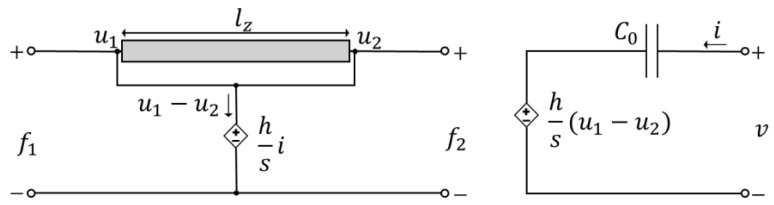
Analogous circuits for the Equations (4)–(6). The controllable voltage sources couple both circuits.

Considering that the electric flow density is constant in the z-axis direction, then dD/dz=0. Therefore, Equations (1)–(3) can be re-written as [19].

(4) $$\frac{d}{dz}\left[f-\frac{h}{s}i\right]=-\rho A_{z}su,$$

(5) $$\frac{du}{dz}=-\frac{s}{A_{z}c}\left[f-\frac{h}{s}i\right],$$

(6) $$v=\frac{h}{s}\left[u_{1}-u_{2}\right]+\frac{1}{sC_{0}}i,$$

where $u_{1}=u\left(0\right)$, $u_{2}=u\left(l_{z}\right)$. $C_{0}=\varepsilon A_{z}/l_{z}$ represents the capacitance between the electrodes of the transducer, and v is the voltage across the transducer’s electrodes. The piezoelectric element can be represented as analogous circuits comprising two parts coupled by controlled sources: an electrical analogous circuit and a mechanical analogous circuit, as shown in Figure 3.

**Figure 4:**
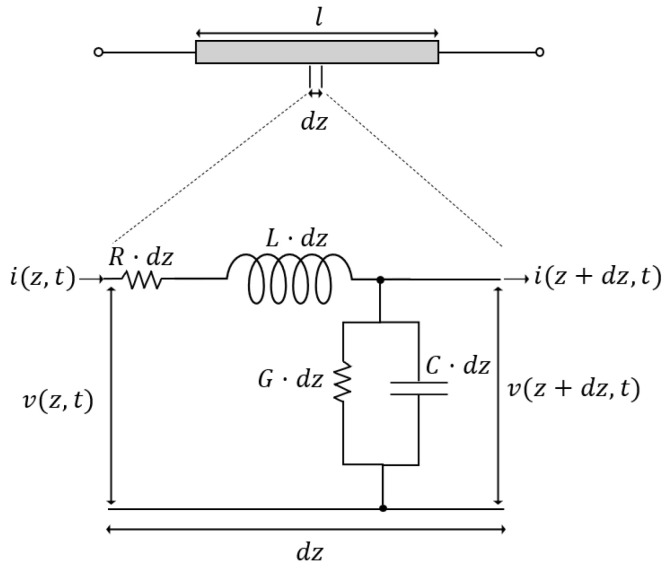
Lumped representation of a transmission line showing its characteristic resistance (R), inductance (L), admittance (G), and conductance (C) for an infinitesimal segment of length dz.

A lumped representation of a lossy transmission line with two conductors, where one of them is a return line, is presented in Figure 4. In this generic representation, the voltage V and current I are governed by the telegraphist’s equations [35,36]:

(7) $$\frac{dV}{dz}=-RI-sLI,$$

(8) $$\frac{d}{dz}=-GV-sCV.$$

In these equations, L, R, G, and C are the inductance, resistance, conductance, and capacitance per unit length, respectively. At high frequencies, the contributions of RI and GV become negligible, and those equations can be re-written as

(9) $$\frac{dV}{dz}=-sLI,$$

(10) $$\frac{dI}{dz}=-sCV.$$

Comparing both sets of Equations (4) and (5) with Equations (9) and (10) yields V=f−hi/s, I=u, $L=\rho A_{z}$, and $C=1/A_{z}c$, demonstrating that a piezoelectric transducer can be treated as a transmission line with two conductors.

### 2.2. Interference in Multiconductor Transmission Line

Once established that a transmission line can be adequately used to model a piezoelectric transducer, the analysis of the interferences inherent to transmission lines with multiple conductors becomes the equivalent of the interaction between two or more piezoelectric patches coupled with the same mechanical structure.

The electromagnetic coupling between conductors in a multiconductor transmission line is usually referred to as crosstalk. The typical cause of crosstalk is due to either poor electrical or mechanical insulation, or both simultaneously. Undesired resistive, capacitive, and inductive coupling in electric circuits and power lines may also cause this type of interference.

**Figure 5:**
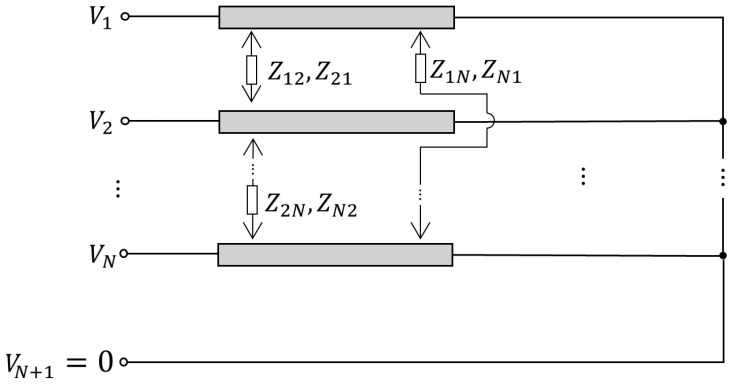
Transmission line with N+1 conductors. $R_{L1}$, …, $R_{LN}$ are load resistances on the line, and $Z_{ij}$ are the mutual impedances between the conductors. The arrows in the mutual impedance elements merely intuitively indicate the direction in which one conductor interferes with the other.

By Ohm’s law, for a given transmission line composed of one conductor and one ground conductor, the voltage $V_{i}^{\prime }$ across it is directly proportional to the current $I_{i}$ flowing in it:

(11) $$V_{i}^{\prime }=Z_{ii}I_{i},$$

where $Z_{ii}$ is a constant of proportionality and is called self-impedance. However, when there are N conductors sharing a common ground, as shown in Figure 5, the crosstalk effect should be accounted for by incrementing $V_{i}^{\prime }$ in terms proportional to the current in the other conductors:

(12) $$V_{i}=V_{i}^{\prime }+\sum_{j=1,j\neq i}^{N}Z_{ij}I_{j}=\sum_{j=1}^{N}Z_{ij}I_{j},$$

where $Z_{ij}$ is the mutual impedance between the i-th and j-th conductors. For this situation, there will be *N* equations which can be arranged in a matrix form:

(13) $$\left\{V\right\}=\left\{Z\right\}\{I\},$$

where $\{V\}={\left\{V_{1}\;V_{2}\;\ldots V_{N}\right\}}^{T}$ and $\{I\}={\left\{I_{1}\;I_{2}\ldots I_{N}\right\}}^{T}\;$ are the voltage and current phasors, and

(14) $$\left[Z\right]=\left[\begin{array}{ccc} Z_{11} & Z_{12}\;\;\;\ldots & Z_{1N}\\ \vdots & . & \vdots \\ Z_{N1} & \ldots & Z_{NN} \end{array}\right]$$

[Z] is the impedance matrix of the transmission line. The elements of the main diagonal are the self-impedances of each conductor (Equation (14)), and the rest are mutual impedances between them. Owing to reciprocity, $Z_{ij}=Z_{ji}$ and [Z] becomes symmetric. Further details on how to obtain an impedance matrix for a transmission line can be found in [37,38].

As the names imply, self-impedance is an intrinsic attribute of a conductor, while the mutual impedance depends on how they are coupled among themselves with the environment in which they exist. Considering purely parallel electrical conductors, the self-impedance can be determined from the telegraphist’s equation as $Z_{ii}=\sqrt{L_{i}/C_{i}}$, and the mutual impedances computed using electromagnetic flux linkage equations [39]. For piezoelectric patches, one must take into account the mechanical coupling (coupling with the host mechanical structure) when calculating the impedances. When there is no host mechanical structure, the piezoelectric transducer is a pure transmission line, and once again, the telegraphist’s equations can be used to determine the self-impedance.

(15) $$Z_{ii}=A_{z}\sqrt{\rho c}.$$

When a host structure is present, the quantity $Z_{ii}=V_{i}/I_{i}\;$ corresponds to the electromechanical impedance of the paired PZT–structure and can be modeled as [8]

(16) $$Z_{ii}=\frac{V_{i}}{I_{i}}=\frac{1}{sa}{\left(\epsilon_{33}^{T}\left(1-j\delta \right)-\frac{Z_{s}}{Z_{s}+Z_{a}}d_{3x}^{2}{\bar{Y}}_{xx}^{E}\right)}^{-1},$$

where $Z_{s}$ is the mechanical impedance of the structure and $Z_{a}$ is the electrical impedance of the PZT. The quantities a, $\epsilon_{33}^{T}$, δ, $d_{3x}^{2}$, and ${\bar{Y}}_{xx}^{E}\;$ are the PZT’s geometric constant, the dielectric constant, the loss tangent, the coupling constant, and Young’s modulus, respectively. A closed formula for determining mutual impedances between two PZTs in a structure has not yet been proposed. However, for the EMI-based methods, it is reasonable to expect that changes to the structural conditions will alter the mutual impedance values. Accordingly, this approach proposes to measure the mutual impedances between a pair of piezoelectric elements attached to a mechanical structure and use those to distinguish changes in the structural conditions.

## 3. The Proposed Method

**Figure 6:**
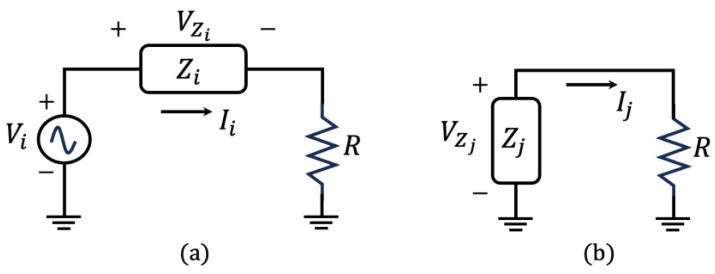
Circuit to compute EMI: (**a**) $Z_{ii}$ and (**b**) $Z_{ij}$, with i≠j and *R* = 1 kΩ.

Based on the above, this paper proposes a new approach in which PZTs are modeled as power transmission lines, allowing us to examine the impedance interactions between PZTs placed close together (at a realistic distance from each other). The method assumes that only one PZT is excited at a time and the others will act only as sensors, with both electrodes grounded. For a given host structure containing N PZT transducers attached to it, by applying an electric potential $V_{i}$ to the i-th exciting PZT, and considering the current $I_{j}^{\left(i\right)}$ generated by the j-th PZT, the matrix presented in Equation (14) can be written as

(17) $$\left[Z\right]=\left[\begin{array}{cccc} V_{1}/I_{1}^{\left(1\right)} & V_{1}/I_{2}^{\left(1\right)} & \ldots & V_{1}/I_{N}^{\left(1\right)}\\ V_{2}/I_{1}^{\left(2\right)} & V_{2}/I_{2}^{\left(2\right)} & \ldots & V_{2}/I_{N}^{\left(2\right)}\\ \vdots & ... & \ddots & \vdots \\ V_{N}/I_{1}^{\left(N\right)} & V_{N}/I_{2}^{\left(N\right)} & \ldots & V_{N}/I_{N}^{\left(N\right)} \end{array}\right]=\left[\begin{array}{cccc} Z_{11} & Z_{12} & \ldots & Z_{1N}\\ Z_{21} & Z_{22} & \ldots & Z_{2N}\\ \vdots & ... & \ddots & \vdots \\ Z_{N1} & Z_{N2} & \ldots & Z_{NN} \end{array}\right],$$

where $Z_{ii}$ are the self-impedances (traditional EMI), and $Z_{ij}$, with i≠j, are the mutual impedances. The main diagonal parameters in Equation (17) are the electromechanical impedances of each PZT determined by Equation (16) (self-impedances in Equation (14)) when a PZT acts as an actuator. The simplified circuits used to determine both impedances are shown in Figure 6, wherein $V_{Z_{j}}$ is the induced voltage due to the direct piezoelectric effect (PZT as a sensor) and $V_{Z_{i}}$ is the voltage for a PZT acting as an actuator.

From Figure 6a, the electrical impedance, which represents the EMI of the set PZT-structure (PZT as an actuator), is given by

(18) $$Z_{ii}\left(\omega \right)=\frac{{V_{Z}}_{i}\left(\omega \right)}{V_{i}\left(\omega \right)-{V_{Z}}_{i}\left(\omega \right)}R\;$$

In Equation (18), one can observe that *R* does not change the shape of the frequency response of the PZT, only acting as a magnitude factor. In fact, the purpose of *R* is to limit the current to avoid loading the PZT. From Figure 6b, the mutual (self-impedance) is computed as follows:

(19) $$Z_{ij}\left(\omega \right)=\frac{{V_{Z}}_{j}\left(\omega \right)}{I_{j}\left(\omega \right)},\;\;\;i\neq j$$

To compute the mutual impedance, only one PZT is excited at a time, and then the induced currents are measured in the others. For example, to compute the mutual impedance of PZT2, the second element of Row 1 in Equation (17), PZT1 is excited and the voltage $V_{1}$ on PZT1 is measured alongside the induced current $I_{2}$ at PZT2. Therefore, $V_{1}/I_{2}$ is the mutual impedance $Z_{12}$ (Equation (14)). A similar procedure is repeated for the next excited PZT and then all EMIs and mutual impedances are computed.

From the matrix presented in Equation (17), it is crucial to obtain a parameter to assess the structure’s conditions. As $V_{i}$ and $I_{j}^{\left(i\right)}$ are in the Laplace domain, the parameter obtained is a function of the frequency and should be equivalent to an impedance (Equation (20)). Since the determinant of a N×N matrix involves products of all elements in the same diagonal/column/row, an Nth power impedance factor will incur after the calculations are fully completed. To retrieve the same impedance order factor, it must apply the Nth root over the determinant of $\left[Z\right]$. The proposed impedance parameter is complex and can be determined as follows:

(20) $$∆Z\left(\omega \right)=\sqrt[{N}]{\det\left(\left[Z(\omega )\right]\right)},\;$$

where the dependency on the angular frequency ω was made explicit.

Once the impedance is a complex parameter, ∆Z(ω) will also be complex and can be analyzed through its magnitude and real and imaginary parts. In the context of SHM systems, researchers predominantly have used the real part of the impedance to identify structural damage [2,3].

Since the objective is only to evaluate the sensitivity of the proposed method, the structure is assessed by comparing ∆Z(ω) in healthy and damaged conditions through the RMSD (root mean square deviation) metric. The RMSD index is based on the Euclidean norm, and it was computed in a specific frequency range, from $\omega_{i}$ (initial frequency) to $\omega_{f}$ (final frequency), as follows:

(21) $$RMSD=\sqrt{\frac{\sum_{\omega_{i}}^{\omega_{f}}{\left[∆Z_{d}(\omega )-∆Z_{b}(\omega )\right]}^{2}}{\sum_{\omega_{i}}^{\omega_{f}}{\left[∆Z_{b}(\omega )\right]}^{2}}},$$

where $∆Z_{b}(\omega )$ is measured in the healthy condition (baseline) and $∆Z_{d}(\omega )$ is the same parameter measured after the structural condition has changed (damaged condition).

## 4. Simulations, Experiments, and Results

This proposed method was analyzed through simulation and experimental tests, initially considering a pristine aluminum beam that was posteriorly damaged by adding mass or applying stress variation. The simulations were carried out on the OnScale^®^ software (Version 1.30.11.0) based on the finite elements (FE) method that allows for complete modeling of PZT and aluminum structure. Experimental tests were carried out on a real aluminum beam. All tests were performed with two PZTs attached to the structure.

### 4.1. Simulations Results

**Figure 7:**
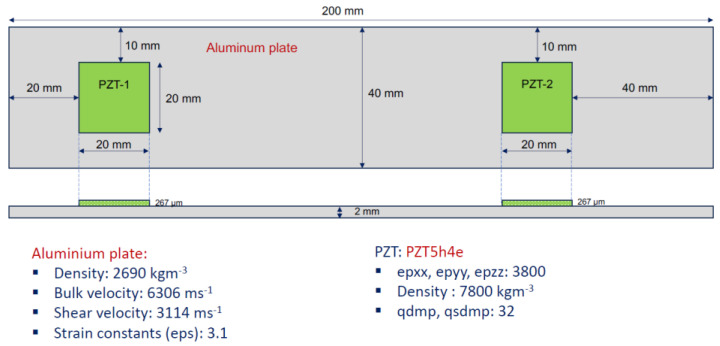
Set PZT–structure and its characteristics—pristine condition.

Figure 7 shows the configuration of the modeled set PZT–structure, including dimensions and material properties.

**Figure 8:**
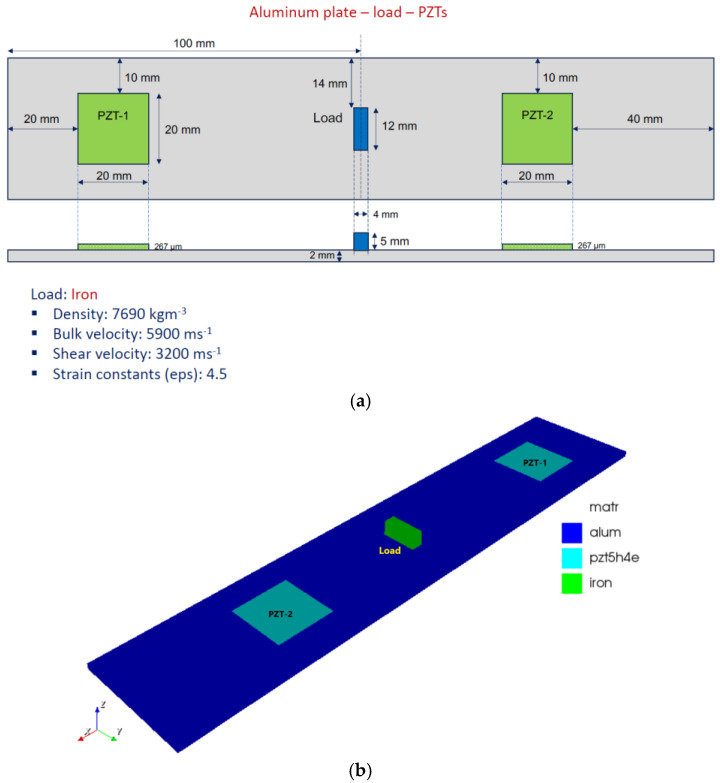
(**a**) Set PZT-structure—damaged condition, and (**b**) OnScale^®^ model.

Figure 8a depicts the simulation setup after the damage was introduced. The damage was simulated by adding a mass named “iron”, positioned between the two sensors, as indicated below. Figure 8b shows the FE model generated using OnScale^®^ software.

**Figure 9:**
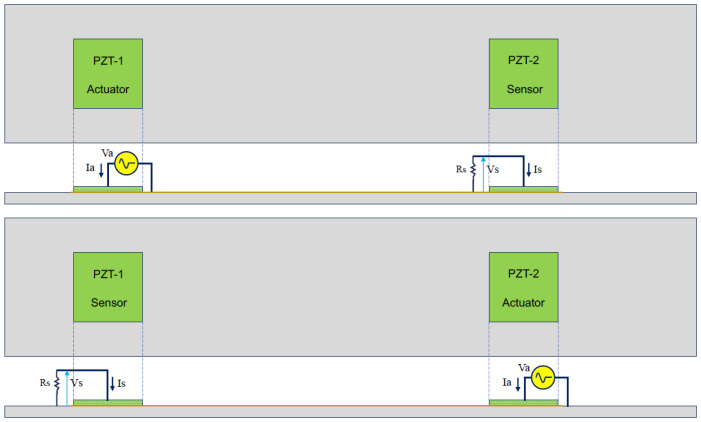
Configuration used for measuring the induced currents.

By nature, the induced currents that generate the crosstalk interference effect are small, and control actions are imperative to avoid charging the PZT sensors. Accordingly, a load resistor was added to the setup in order to correctly compute the mutual impedances (PZTs acting as sensors). The configurations adopted for both cases (different PZTs acting as sensors and actuators) are presented in Figure 9. It can be observed that the PZTs have the same reference, as specified in the proposed model.

Several tests were carried out to analyze the influence of the load resistor ($R_{s}$). It was found that values varying from 1 kΩ to 1 MΩ can be utilized without changing the impedance curves. The results here obtained considered $R_{s}$ = 1 MΩ, leading to high mutual impedance. The excitation signal comprises a finite duration pulse with an amplitude of ±2 volts, which is similar to the spectrum of a chirp signal in a frequency range from 1 kHz to 300 MHz. It is important to highlight that this type of signal is available in OnScale^®^ and adequately replaces a chirp-type signal, typically used in practical tests. The FE simulations were performed using a box-type structured grid, 15 elements per wavelength, an element size of 1.3 mm, and a simulation time of 30 ms. For simulation purposes, the boundary conditions for the beam were set as free–free.

**Figure 10:**
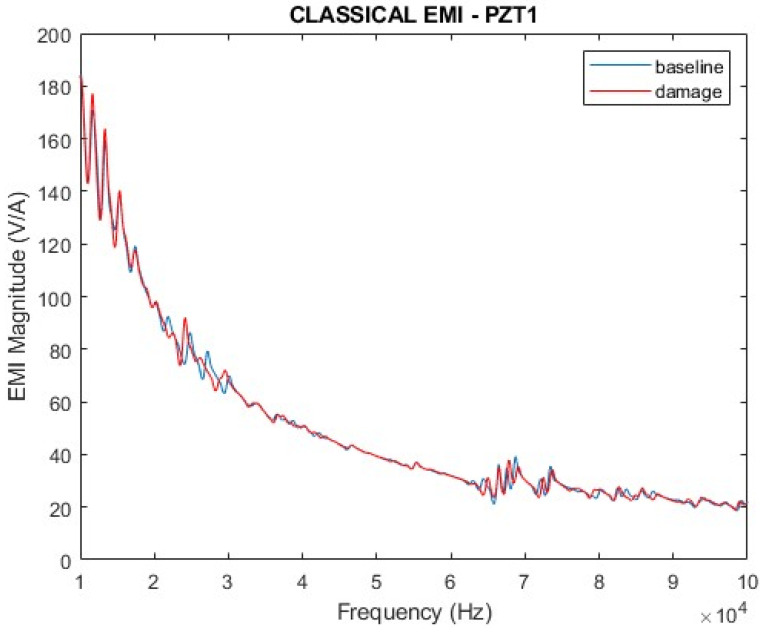
Magnitude of the electromechanical impedance—PZT1 (simulation).

**Figure 11:**
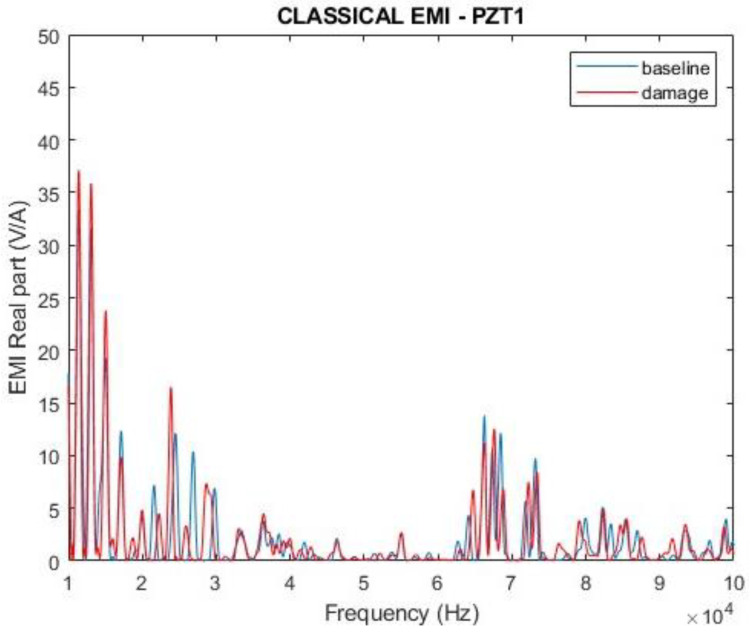
Real part of the electromechanical impedance—PZT1 (simulation).

**Figure 12:**
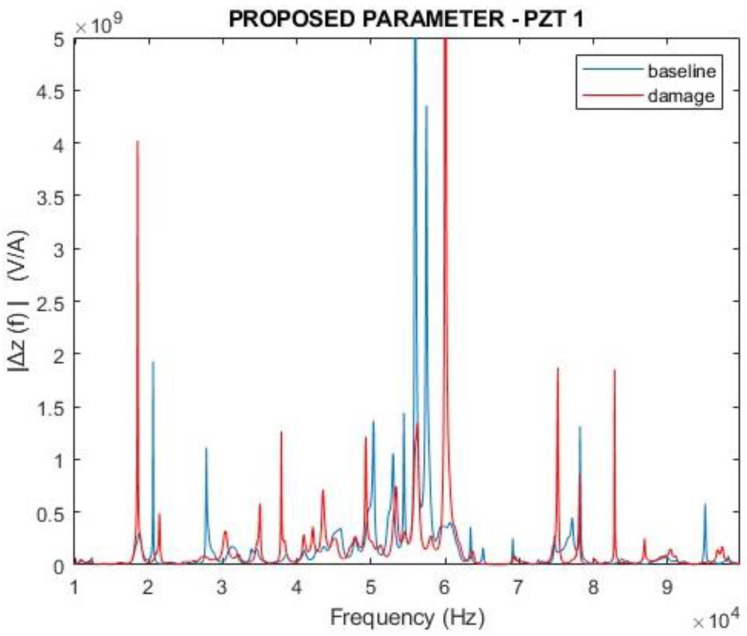
Magnitude of the proposed parameter ∆Z(f) for PZT1 (simulation).

**Figure 13:**
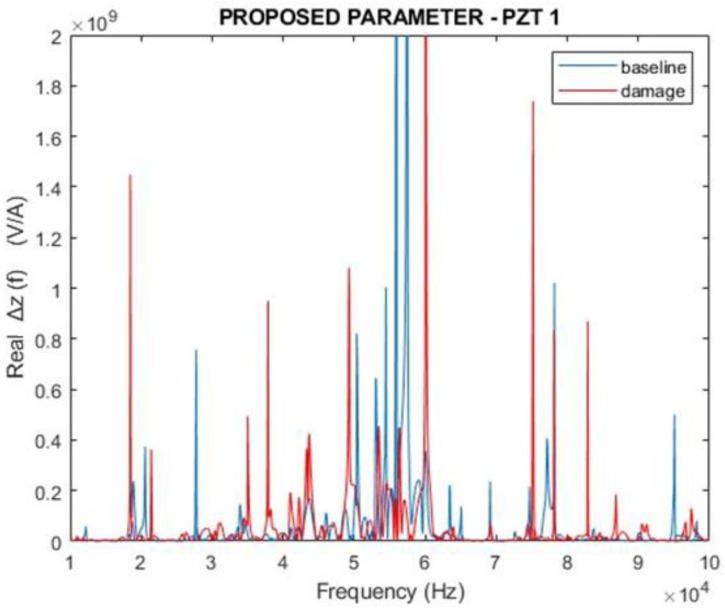
Real part of the proposed parameter ∆Z(f) for PZT1 (simulation).

The results for the original impedances (indicated in Equation (16)) and for the parameter proposed in Equation (20) are presented in Figure 10, Figure 11, Figure 12 and Figure 13 for a frequency range from 10 kHz to 100 kHz. Since PZT1 and PZT2 obtained similar results, only the ones obtained from PZT1 will be presented hereafter. Figure 10 and Figure 11 show the magnitudes and the real parts of the traditional EMI for PZT1, obtained in healthy (baseline) and damaged conditions. The EMI signatures are like the typical ones encountered for an aluminum plate according to the findings presented in the literature [1,40]. It can also be observed that, in both cases, it is possible to clearly differentiate the impedance signatures in healthy and damaged conditions.

Figure 12 and Figure 13 depict the magnitudes and real parts of the ∆Z(f) parameter proposed in this work for f=ω/2π (Equation (20)). Similarly, the analysis took into consideration both healthy (baseline) and damage scenarios. The results are expressive and show how different the curves are.

Compared with the curves presented in Figure 10 and Figure 11, there is no doubt that the proposed parameter presents higher variations in the damage condition than the traditional EMI. Furthermore, one can observe that the response of ∆Z(f) indicates differences between the baseline and damage curves in the entire analyzed frequency band, whilst the traditional EMI shows poor sensitivity in the range of 40 to 65 kHz. Indeed, the sensitivity of these frequency ranges depends on the characteristics of the structure and should be analyzed case by case.

**Figure 14:**
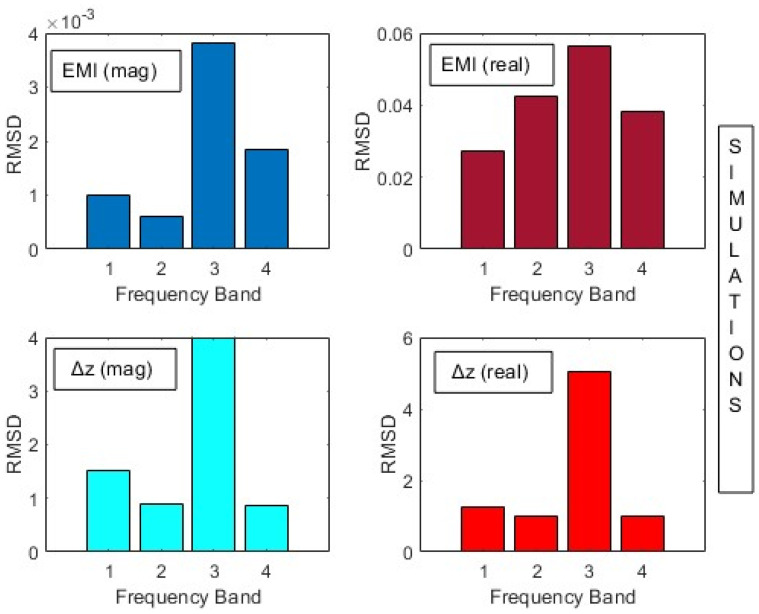
RMSD indexes for traditional EMI and the proposed method (simulation).

A better comparison between the proposed method and the traditional EMI was conducted by computing the RMSD index for both the magnitude and real part (Equation (21)). Owing to the difference in sensitivity per frequency band as mentioned before, the indices were computed in four distinct bands (Band 1: 1–30 kHz; 2: 30–60 kHz; 3: 60–90 kHz; 4: 90–120 kHz). These results are shown in Figure 14. It is noteworthy to highlight that these bands were assessed in both simulations and practical tests because they contain the most important variations between healthy and damaged conditions for both EMI and the proposed parameter.

In both cases, the RMSD indexes indicate the existence of damage. Band 3 presents higher RMSD metrics, allowing better damage detection for the present structure. It is important to note that the values of mutual impedances are many orders of magnitude greater than those of traditional EMI. This means the proposed method is more sensitive and easier to implement in real SHM systems, since the margin for defining a decision threshold between healthy and damaged conditions is wider. Another remarkable observation from Figure 14 is that the RMSD indexes obtained from the magnitude and real part of ∆Z(f) present similar variations for all bands, which is not the case for the EMI. Either way, the real part shows higher RMSD indexes in both cases.

### 4.2. Experimental Results

**Figure 15:**
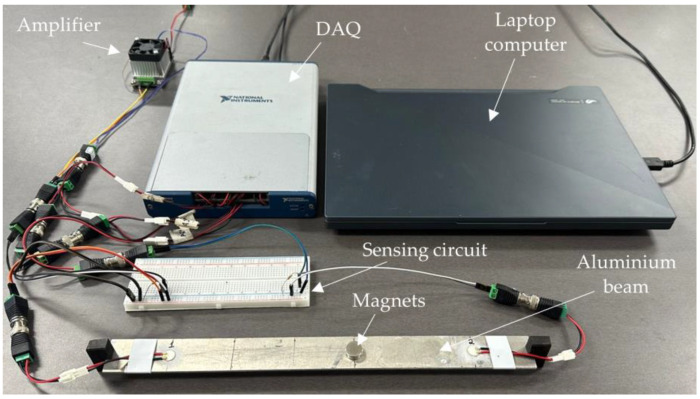
Test bench used for the experimental tests.

The simulations were fundamental to evaluating the effectiveness of the proposed method. Notwithstanding, considering the wide range of practical applications of EMI-based techniques and the low currents involved in the proposed method, experimental tests play a key requirement in demonstrating the feasibility of the proposed method. Accordingly, an experimental apparatus was set up consisting of a thin aluminum beam of dimensions 450 × 30 × 2 mm supported at both ends. Two PI PRYY + 0226 circular piezoelectric patches (∅ 10 × 0.5 mm) were attached to the beam and placed near its ends (350 mm apart from each other). The damage was simulated by using two magnets with a 15 mm diameter aiming to change the mass and stress states of the plate. This experimental setup is depicted in Figure 15.

**Figure 16:**
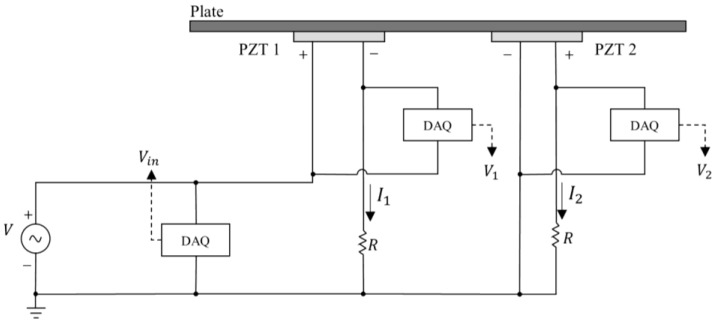
Measurement circuit used to measure impedances.

The schematic of the circuit used for measuring the impedances, when PZT1 was used as actuator, is shown in Figure 16. A National Instrument USB-6366 DAQ (National Instruments Corporate Headquarters, Austin, TX, USA) was used to generate the excitation signal via a PiezoDrive PDu150 amplifier (Piezo Drive, Shortland, NSW, Australia) and to measure the exciting voltage ($V_{in}$) and the voltage across the PZTs ($V_{1}$ and $V_{2}$). This configuration is based on the low-cost circuit presented in [41]. This setup was used to measure $Z_{1j}$, the first line of the matrix [Z]. To measure the second element $Z_{2j}$ of the impedance matrix (Equation (17)) when PZT2 is excited, the voltage source V is connected to PZT2 and this procedure is repeated. The excitation signal considered here was a sine chirp from 1 kHz to 250 kHz and the signals were sampled by the DAQ at a rate of 1 MHz. Finally, the measured voltages $V_{1}$ and $V_{2}$ are used to determine the currents $I_{1}=(V_{in}-V_{1})/R$ and $I_{2}=V_{2}/R$ (PZT1 excited). The impedances were posteriorly calculated using Equation (17). A laptop was also used to control the tests and process the results. For example, for a 2 × 2 matrix (2 PZTs) for the experiment setup shown in Figure 16, the magnitude of Z is obtained by

(22) $$\left[Z\right]=\left[\begin{array}{cc} \frac{V_{1}}{V-V_{1}}R & \frac{V_{1}}{V_{2}}R\\ \frac{V_{2}}{V_{1}}R & \frac{V_{2}}{V-V_{2}}R \end{array}\right].$$

Indeed, as presented before and indicated by Equations (18) and (19), all voltages and impedances are frequency dependent, which means that all elements of the matrix are vectors in frequency.

**Figure 17:**
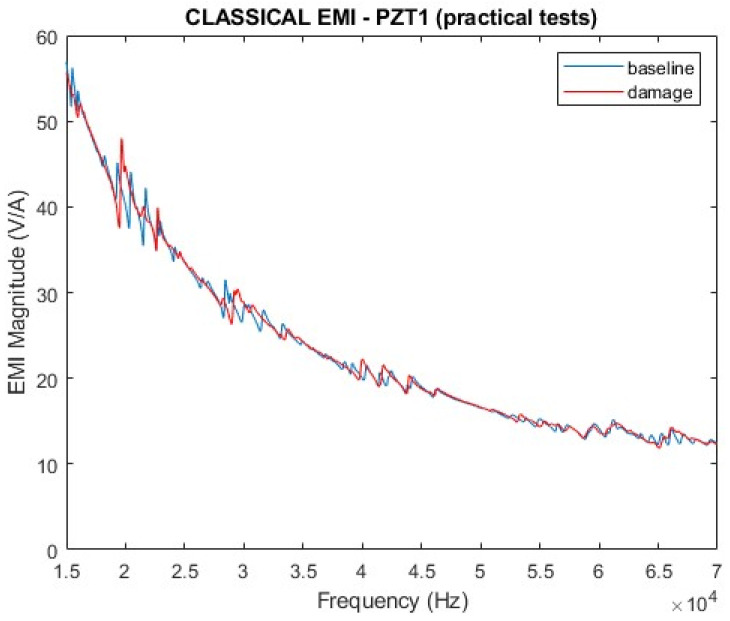
Magnitude of the EMI for PZT1 (experimental).

**Figure 18:**
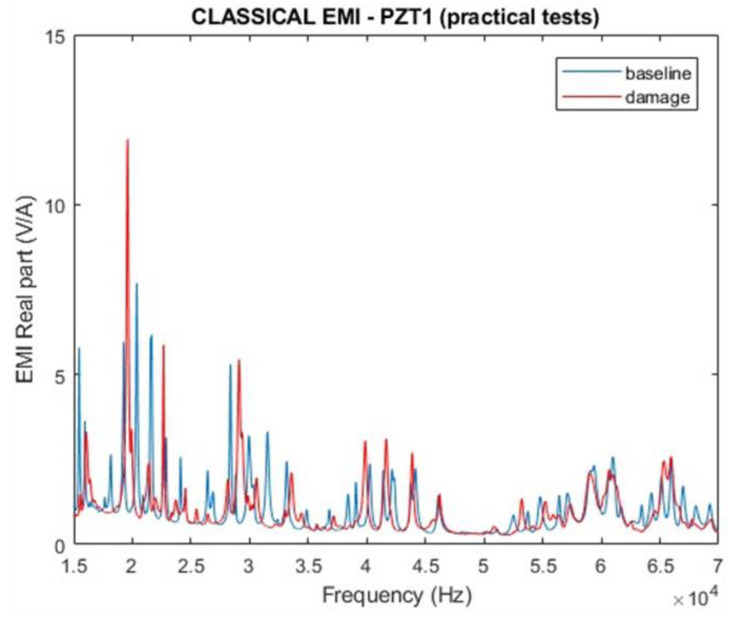
Real part of the EMI for PZT1 (experimental).

After certifying that the EMI signatures for PZT1 and PZT2 are similar, only those results obtained from PZT1 are presented. Figure 17 and Figure 18 show the EMI signatures for PZT1, including the magnitude and real part. All results have considered healthy and damaged structural conditions in a range of frequencies from 15 kHz to 70 kHz. In both cases, it is possible to differentiate the impedance signatures between normal (baseline) and damaged states.

**Figure 19:**
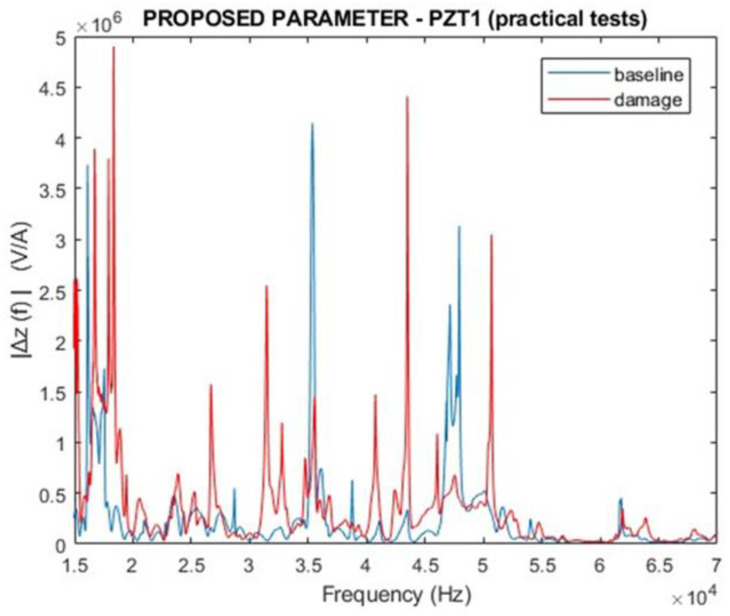
Magnitude for the proposed parameter ∆Z(f) for PZT1 (experimental).

**Figure 20:**
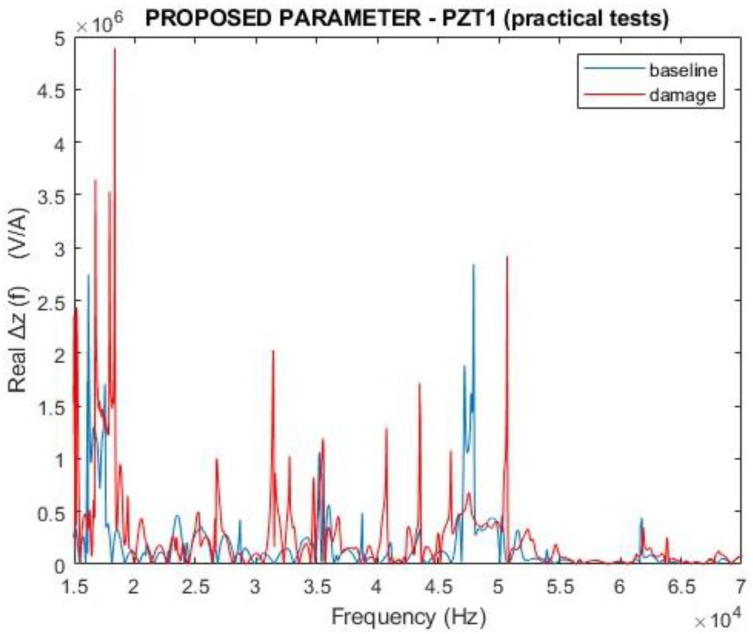
Real part of the proposed parameter ∆Z(f) for PZT1 (experimental).

Figure 19 and Figure 20 present the results of the proposed parameter ∆Z(f), considering the magnitude and the real part. The results demonstrated how different the two curves are. Compared with the curves presented in Figure 17 and Figure 18, the results for the proposed parameter demonstrated higher variations in the damaged condition than traditional EMI.

**Figure 21:**
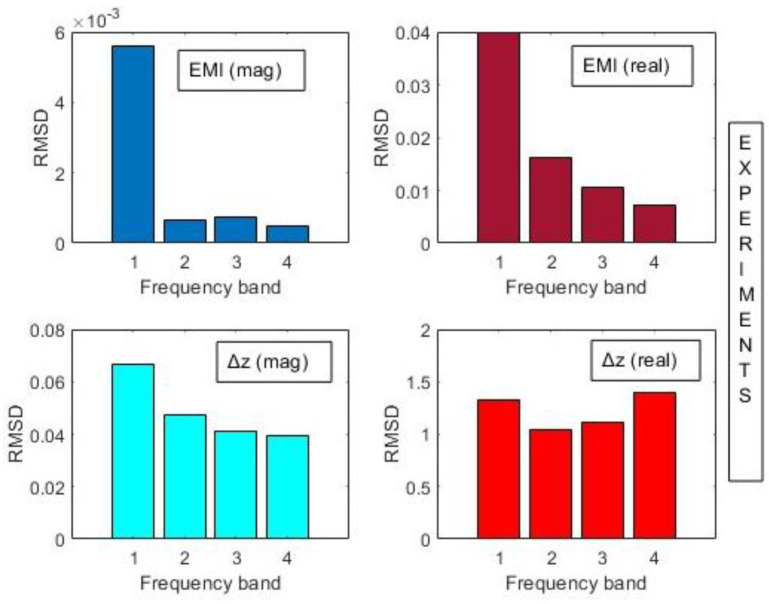
RMSD indexes for traditional EMI and the proposed method (experimental).

Finally, the RMSD indexes were computed for the magnitudes and real parts for both methods within the frequency bands for Band 1: 1–30 kHz; 2: 30–60 kHz; 3: 60–90 kHz; and 4: 90–120 kHz. These results are presented in Figure 21. The results are consistent with those obtained from simulations and confirm the effectiveness of the proposed method. It is noteworthy to mention that the results for the proposed method were more constant across the frequency bands, showing that any band could be used to set a threshold for damage detection. For the traditional EMI, the first band demonstrated higher indices for damage detection.

The experimental results demonstrated the effectiveness of the method and endorsed the simulations. Comparing the simulations and experimental results, one can ascribe that the main differences are related to the magnitude of ∆Z(f). This is due to using different load resistances to measure the current across the PZT (1 MΩ for simulations and 1 kΩ for experimental tests). This difference is also associated with the dimensions of the plate and boundary conditions. Considering that the magnitudes of the estimated mutual impedances are huge, it implies that the measured currents are tiny. Therefore, any instrument used for the measurements would cause a loading effect, because of its input impedances. Although very high, it will not be comparable with the magnitudes of the sought impedances. Thereupon, the measurements could be adjusted/corrected according to the technical information of the data acquisition device.

## 5. Conclusions

This paper proposed a new approach to detect structural damage based on the electromechanical impedance by considering the crosstalk effect in arrangements of PZTs modeled as a multiconductor transmission line. That effect was measured through the coupled electromechanical impedance matrix obtained from modeling the set PZT–structure as a transmission line system. Experimental tests were carried out in an aluminum beam structure containing two attached PZT transducers. Simulations using finite element modeling on the OnScale^®^ software were also conducted. For both approaches, the structure’s healthy and damaged conditions were considered. The results were compared with those obtained using a traditional EMI-based method and proved that the new approach significantly improves the sensitivity of the EMI-based techniques in SHM applications. It is worth pointing out that any practical implementation of the proposed method must consider all drawbacks inherent to EMI-based techniques, such as temperature variation, the glueing effect on the PZTs, and boundary conditions.

## Data Availability

Data are contained within the article.
